# High-Entropy Alloys Produced by Mechanical Alloying: A Review

**DOI:** 10.3390/ma19071300

**Published:** 2026-03-25

**Authors:** Jason Daza, Asma Wederni, Rehan Ullah, Joan Saurina, Lluisa Escoda, Joan-Josep Suñol

**Affiliations:** Department of Physics, Universitat de Girona, P2, EPS, Campus Montilivi s/n, 17003 Girona, Spain; jason.dazac@udg.edu (J.D.); asma.wederni@udg.edu (A.W.); rehan.ullah@udg.edu (R.U.); joan.saurina@udg.edu (J.S.); lluisa.escoda@udg.edu (L.E.)

**Keywords:** high-entropy alloy, mechanical alloying, powder metallurgy, mechanical properties, magnetic properties

## Abstract

High-entropy alloys formed by metals are usually classified as those with magnetic elements, such as Fe, Co, and Ni, and alloys containing a significant percentage of aluminum. In the first case, the functional responses of greatest scientific and technological interest are both mechanical and magnetic. Concerning applications, the main interest focused on health and energy. Among the various techniques used to obtain high-entropy alloys in powder form, one of the most widely applied is mechanical alloying. This paper reviews recent results and prospects, including machine learning.

## 1. Introduction

### 1.1. Mechanical Alloying

Mechanical alloying (MA) is a solid-state synthesis technique that involves the mechanical grinding of powdered precursors (or chips) to produce powder particles [1]. The multiple and intense mechanical collisions and friction between the grinding media (containers and balls) and the powder typically result in the formation of an alloy at the atomic level. Milling is considered high energy when the kinetic energy of the balls is high during ball–powder–ball or ball–powder–container collisions. This is achieved by intense mechanical collisions and friction between the grinding media and the powder. Due to the repeated welding and fracturing of powder particles, the formation of alloys and compounds, with the refinement of their structure, occurs over time [2].

The existing milling equipment at the laboratory level is of the planetary or shaker type. In planetary milling machines, the containers with the powder rotate in one direction on a platform that rotates in the opposite direction. A characteristic parameter of processing by mechanical alloying is the milling frequency or intensity. The higher the frequency or intensity, the greater the energy and power transferred to the powder. At low frequencies/intensities, the main process is abrasion; at high frequencies, it is percussion. As the power or energy transferred increases, the density of crystallographic defects increases. To avoid oxidation processes on the alloys, milling is carried out under an inert argon or nitrogen atmosphere. One of the problems that milling often entails is contamination from the milling medium (containers and balls) due to continuous friction. Therefore, a compositional analysis of the alloys must be carried out a posteriori.

Another relevant parameter is milling time. The mechanical alloying process can be grouped into several stages depending on the specific material: the initial mixing stage of the precursors, a period of predominant welding or fracture, and an ending in a steady-state situation. The different stages can be explained in terms of (a) the powder distribution and shape, (b) how hard the material is at the ball surface, (c) the microstructure of the powder and material at the ball surface, and (d) the partitioning of the material between the ball surfaces and the free powders [3]. Abrasion and percussion increase the local temperature of the powders. As a consequence, the overall temperature inside the container also increases. To avoid excessive overheating, it is common to program a duty cycle (turning the milling device on and off).

It is normally recommended that containers are filled between 50 and 67% of their volume with precursor particles and balls. One parameter provided when describing grinding is the ball-to-powder weight ratio (BPR) [4]. Typical values reported in the literature are 5:1 or 20:1. This parameter is important so that abrasion and percussion phenomena involve a high percentage of powder particles without a high filling percentage acting as a brake, preventing the balls from achieving high kinetic energy. Thus, a high BPR is associated with increased energy transferred to the particles. Regarding ball size, they can be identical or of two different sizes to facilitate interactions in curved areas of the container. To analyze microstructural evolution throughout the milling process, intermediate extractions are often performed. This fact, combined with the variation in average particle size during milling, means that the filling percentage or BPR ratio is not constant.

Depending on the brittle or ductile behavior of the particles, cold welding or their fracture during the grinding process is possible. If the smallest possible particles are desired, a process control agent (PCA) is introduced, which hinders excessive cold welding and particle agglomeration, facilitating the generation of free surfaces [5]. These allow elemental diffusion phenomena and alloy formation. PCAs are liquids or solids: methanol, ethanol, isopropyl alcohol, heptane, hexane, cyclohexane, polyethylene glycol, stearic acid, sodium chloride, and so on. An additional effect of the PCA is the diminution of the crystallite size.

Other important aspects that limit the applicability of the material produced by mechanical alloying are the shape and size of the particles. The particles are usually micrometer-sized, and although rounded, their specific surface area is relatively high. Regarding industry, the main limitation is the transition from laboratory to industrial scale. Also, maintaining microstructure and good functional properties after powder consolidation when obtaining the bulk remains difficult.

**Figure 1:**
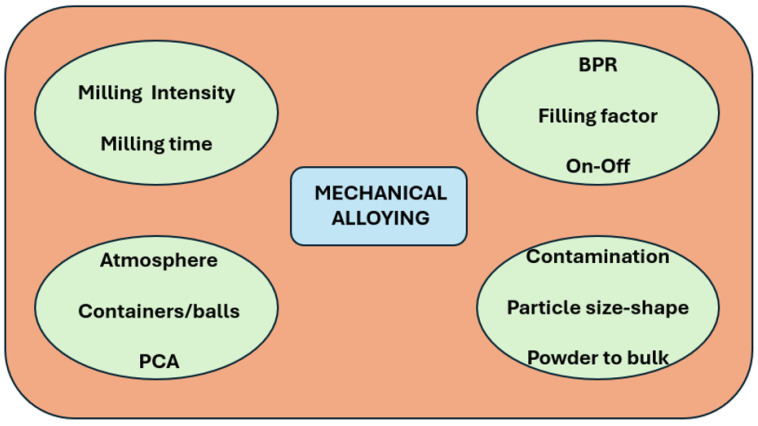
The schema of mechanical alloying’s main parameters linked to the devices, the milling media, the control of the process, and the main technological issues. BPR: Ball-to-powder weight ratio. PCA: Process control agent.

Figure 1 shows a diagram of the various parameters and aspects to be taken into account in mechanical alloying associated with the device parameters (milling intensity and milling time), the milling media (atmosphere, PCA, and containers–balls material), some processing parameters (BPR ratio, filling factor, and time on–off), and several technological issues (such as contamination, the need for consolidation to produce bulk pieces, and the particles’ size distribution and shape). All these aspects also affect the use of this technique in the production of high-entropy alloys (HEAs).

### 1.2. High-Entropy Alloys

In recent decades, the production and structural and functional characterization of so-called high-entropy alloys has been a growing field. The most widely accepted definition currently is that they are alloys with at least five elements, which all have a percentage between 5% and 35% [6,7].

As multicomponent systems, they are characterized by atoms with different atomic radii, diverse equilibrium crystal structures, and diverse bond energies. Their varying sizes hinder the movement of dislocations that occur during the mechanical alloying process, facilitate distortion of the crystal lattice, and lead to solution strengthening. When five or more elements are present, synergies occur due to the interaction between the various elements or the intermediate products obtained, some of which are complex to understand. All of this is associated with the cocktail effect. Another result is that diffusion rates are different with the slowest ones dominating overall processing. Therefore, the formation of nanocrystalline structures is facilitated, the particle coarsening rate is reduced, and the recrystallization temperature is increased. The main characteristic is its high entropy. High entropy hinders the final formation of intermetallic compounds and instead favors the formation of a single solid solution. The main characteristics, denominated as core effects, of these alloys are generally considered to be high entropy, lattice distortion, sluggish diffusion, and the cocktail effect [8].

It has been found that HEAs form different crystal structures as solid solutions: face-centered cubic (FCC), body-centered cubic (BCC), hexagonal close-packed (HCP), amorphous alloys, or eutectic mixtures.

**Figure 2:**
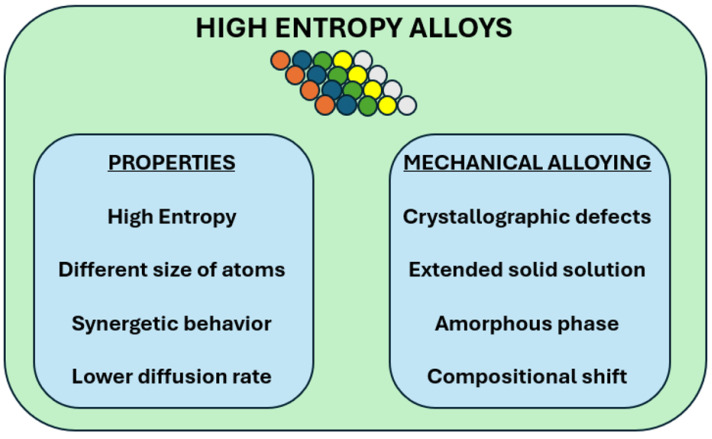
Schema with high-entropy alloys’ characteristics and the main effects that MA can provoke: crystallographic defects, amorphous phases, extended solid solutions, compositional shift.

Figure 2 shows a schematic representation of the main characteristics of high-energy alloys and the aspects related to mechanical alloying. Mechanical alloying favors the formation of extended solid solutions and metastable phases. It also favors a high density of crystallographic defects and unwanted contamination of the milling medium.

To characterize, classify, and/or sort the various shape memory alloys, there are multiple parameters. Table 1 shows a list of HEA criteria applied in the scientific literature [9,10]. Diagrams are also constructed by combining the values of two parameters, such as the Darken–Gury plot, in which electronegativity is represented versus the difference in atomic size.

There are several classifications of high-entropy alloys. Most of them are linked to the periodic table of elements. One classification [11] is (a) FCC strong and ductile HEAs: elements such as Cr, Mn, Fe, Co, and Ni; (b) BCC refractory HEAs: elements such as Ti, V, Zr, Nb, Mo, Hf, Ta, and W; (c) HCP HEAs: elements such as Y, Gd, Tb, Dy, and Ho; (d) lightweight HEAs: elements such as Ti, Li, Be, Mg, Sc, and Al or (e) precious functional HEAs: elements such as Ru, Rh, Pd, Ag, Ir, Pt, and Au.

Another (similar) classification is based on the chemical nature of most of the constituent elements [12]. In this case, HEAs are classified into four main families: (a) 3d transition metal high-entropy alloys (3d TM HEAs), constituted by Fe, Ni, Co, Mn, Ti, and Cr, among others, typically exhibiting face-centered cubic (FCC) disordered solid solutions and corresponding to the most explored subfamily of HEAs; (b) refractory high-entropy alloys constituted by elements of the groups IVB, VB, and VIB, commonly presenting disordered body-centered cubic (BCC) solid solutions; (c) low-density high-entropy alloys, regularly crystallizing into hexagonal closed-packed (HCP) solid solutions and constituted by light elements like Al, Be, Li, Mg, Ti, and Sc, among others; and (d) high-entropy alloys constituted by at least four of the lanthanide elements, also exhibiting HCP solid solutions.

Additionally, this review also explores the promising applications of HEAs [13]. The future research directions focus on the interplay between multi-scale structures and properties, the innovative production technologies, and the sustainable alloy recycling and reuse strategies. 

Concerning the influence of the elements, HEAs based on Fe, Co, and Ni favor magnetic behavior. Co, Ni, Mn, and Ti additions improved the resistance to corrosion. Cu and Ni additions favor the formation of the FCC phase, whereas the Al and Ti additions favor the BCC phase. Likewise, the increase in Co content improved both the compressive strength and fracturing strain. The Ni content shifted the slip mode from planar to wavy, leading to microband formation and a substantial enhancement in plasticity.

### 1.3. Mechanical Alloying of High-Entropy Alloys

The research on HEAs will take into account several phenomena, improving the main properties and core effects of these alloys. The nanoscale elemental mixing during milling enhances the high entropy effect and the generation of crystallographic defects. Furthermore, the mechanically induced plastic deformation increases the lattice distortion, while the homogeneous and uniform element distribution at the nanoscale favors the cocktail effects, and the generation of free surfaces during milling enhances the sluggish diffusion near room temperature. Nevertheless, there are some aspects to be checked, such as the compositional shift and media contamination during milling, the induction of undesired amorphous phases, and the issues linked to the microstructure and property changes produced during the consolidation and annealing (solid-state phase transformation, such as metastable phase decomposition).

There are several aspects to consider in high-entropy alloys produced by mechanical alloying, contributing to the different mechanical and functional responses when comparing them with HEAs produced by other techniques [14,15]. They are related to each other.

(a) Mechanical alloying favors the formation of metastable, non-equilibrium phases. Therefore, it allows the production of phases not expected from a thermodynamic point of view. These can be intermetallic compounds (at short and medium milling times), nanocrystalline phases (with a high percentage of atoms at grain boundaries), amorphous (disordered) phases, or supersaturated solid solutions (exceeding the solubility limits expected according to equilibrium phase diagrams or in systems with a positive enthalpy of mixing).
(b) Mechanical alloying favors a high density of crystallographic defects, provoking a high lattice distortion. The distortion is higher than in HEAs produced by other techniques. A high density of crystallographic defects results in faster atomic transport during heat treatment. Likewise, the high defect density (dislocations, vacancies, and grain boundaries) increases the storage energy (an additional term due to these defects), thereby shifting the equilibrium. High-energy grain boundaries become unstable. As the milling time increases, the increasing lattice strain (defect accumulation) acts as the driving force for the amorphous phase formation.
(c) Contamination usually comes from the milling tools (mainly iron, as steel is the most used material for balls and containers), the atmosphere (oxygen), or the PCAs (usually carbon). Sometimes, oxides or carbides are formed. It should be remarked that interstitial strengthening from the oxygen and carbon contamination provokes the enhanced hardness of the HEAs produced by MA. Carbon from common PCAs (such as stearic) often forms in situ carbides that significantly boost hardness but plummet ductility. On the other hand, contamination can act as a stabilizer of a specific crystallographic phase. For example, in Ti-containing HEAs, oxygen contamination can trigger the formation of undesired TixOy oxides or stabilize an HCP phase.

Likewise, there are several key problems associated with the mechanically alloyed HEAs [16,17].

(a) Surface/volume ratio: The powdered particles ranged in the micrometric range, and the continuous fracture and welding favor a relatively high surface/volume ratio. In compounds with metallic elements, the surface oxidation occurs when particles are in contact with the atmosphere.

Solution/mitigation: milling in an inert atmosphere, such as argon. Manage the powdered samples in a glove box.

(b) Milling tools: The continuous shocks and abrasion with the balls and the inner surface of the containers provoke contamination from the milling tools, shifting the final composition with respect to the original one. One typical contamination is iron from the steel balls and containers.

Solution/mitigation: reducing the milling intensity; working with milling media that are wear-resistant.

(c) Milling time: The production of a unique phase in multicomponent alloys is obtained after long processing times.

Solution/Mitigation: increase the milling energy to reduce time. However, it can induce more contamination from milling tools.

(d) Phase homogeneity and metastable phases: High milling times are needed for phase homogeneity. Furthermore, at low milling times, metastable phases such as intermetallic compounds can be formed.

Solution/mitigation: One option is ulterior annealing to homogenize the compound.

(e) Agglomeration: Dry milling favors the formation of agglomerates and the consolidation of these on the surface of the inner walls of the containers.

Solution/mitigation: Milling using a process control agent acting as a lubricant. Sometimes, contamination is produced.

(f) Microstrain and crystallographic defects: The continuous milling provokes an increase in the microstrain and the density of crystallographic defects.

Solution/mitigation: Ulterior thermal treatment facilitates the structural relaxation.

(g) Irregular shape: The irregular shape and non-spherical shape after fracturing-welding provoke low flowability for additive manufacturing processes.

Solution/mitigation: Further treatment by laser spheroidization to obtain spherical particles. It can induce microstructural changes.

(h) Consolidation: The powdered samples should be compacted to obtain bulk alloys. The main issue is the final density achieved, which is usually lower than the theoretical one.

Solution/mitigation: consolidation under high pressure; ulterior thermal annealing or sintering.

(i) Lab to industrial scale: The experiments in the laboratories produced low amounts (several grams) of the alloys. The scaling up to industrial application is complex. There are so many processing parameters involved.

Solution/mitigation: Modeling the scale influence of the scale parameters. Analyzing the energy effect due to heating and the final homogeneity of the produced compounds.

Concerning processing conditions for high-entropy alloys, one of the important parameters is milling time. A longer milling time is typically required for high-entropy alloys (HEAs) to achieve a single crystallographic phase. At short milling times (up to 20 h), there are metastable phases and incipient solid solution phases. Increasing the time to 50 h facilitates the development of the predominant phase, while milling times exceeding 50 h (up to 200 h) are necessary to obtain homogeneous single-phase alloys.

Another parameter is the ball-to-power weight ratio. High BPR values favor the fracture regime as the dominant process, while low values favor the cold-welding regime. The energy per unit mass transferred to the powder particles during the milling process is considered directly proportional to the BPR value. A typical BPR ranges between 5:1 and 20:1.

With respect to the PCA, a quantity of PCA on the order of milliliters is added with 1 mL being the most common amount in containers up to 80 mL. Regarding the weight percentage, the studies suggest the addition in the range 1 to 3 wt.%. There is a compromise between modifying the fracture-cold welding equilibrium and the contamination caused by the addition of PCA.

The rotational speed is related to the energy transferred per unit mass. In planetary grinding equipment, speeds up to 200 rpm correspond to a regime dominated by wall–powder or ball–powder collision processes. Above 400 rpm, collisions predominate, and at intermediate speeds, both regimes coexist. The high energy impacts in a ball milling device generate a dislocation density achieving values such as 10^16^ m^−2^. Moreover, due to the high-energy impacts, the residual microstrain in MA-HEAs is typically in the range 0.6 to 1.5%.

Regarding the thermal annealing, it should be customized to every MA specimen. If the annealing/sintering temperature is too high, the induced grain growth modifies the benefits of the nanocrystalline size. Likewise, processes such as the formation of precipitates or the phase separation/decomposition diminish the mechanical toughness. Table 2 shows the influence of several MA parameters on the microstructure and on the HEA specimens.

A comparison of the same features between MA, cast methods, and additive manufacturing is given in Table 3.

There are also issues linked to the thermodynamics and kinetics of the HEAs produced by MA.

Thermodynamics: MA forces the elements’ solubility into a single-phase solid solution (SS) by overriding positive enthalpies of mixing (with sheer mechanical energy). Ulterior annealing/sintering provides thermal activation for the alloy to modify the microstructure. The main parameters are the entropy and enthalpy. It is a competition between both parameters. Milling at low intensity (thus, low temperature), the configurational entropy cannot overcome a positive mixing enthalpy. It is necessary to sinter at a sufficient temperature to enhance the entropy to values that permit the atomic motion and the formation of metastable phases. Another parameter is Gibbs energy; the reduction in the crystallite size to the nanoscale produces an increase in the surface/volume ratio. Annealing reduces this ratio (crystallite growth), favoring solid-state transformations such as phase decomposition. The transformation stops crystalline grain growth.

With respect to kinetics, the induced high defect density promotes atomic motion, thereby decreasing the effective activation energy for solid-state transformation. Moreover, thermal scans also show broad exothermic processes at low temperatures associated with the structural relaxation of the MA powders. Calorimetric scans are typically employed to detect these relaxation effects and activation energies of the solid-state transformations [21]. Another difference with HEAs produced by other techniques is that MA facilitates nanoscale diffusion (atoms need to move only a few nanometers to form a nucleus) as an alternative to sluggish diffusion.

Concerning the phase stability, the grain boundaries increase the interfacial energy, the dislocations increase the lattice strain energy, the vacancies facilitate the mass transport and diffusion, and the anti-site defects facilitate the chemical disorder.

## 2. Experimental Approach

### 2.1. Particles Characterization

**Figure 3:**
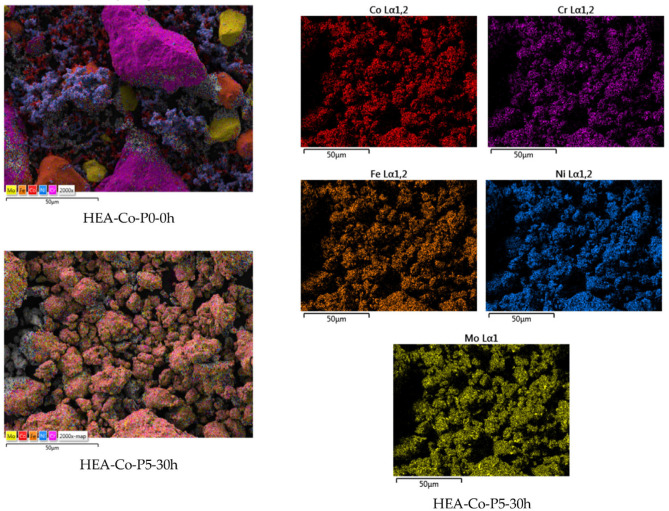
Elemental mapping analysis results for two Cr-Fe-Ni-Mo-Co HEAs under different conditions. HEA-P5-30h sample alloyed for 30 h compared to the HEA-Co-P0-0h homogenized sample [22].

The mechanically alloyed alloys and compounds require characterization. The powders (in the micrometric range) are characterized to determine the particle size distribution and shape, the crystallographic structure, the thermal stability, and the expected functional response. One option to observe the particle size and shape is scanning electron microscopy (SEM). Since these are multi-element systems, the homogeneity of the alloys must be verified with microanalysis. One option is elemental mapping, which verifies whether all the elements are detected in the different particles. An example of elemental mapping is shown in Figure 3 [22]. It is also recommended to have knowledge of the particle size distribution. The dynamic light scattering (DLS) technique is an optimal procedure [23]. Another property is the surface-to-volume ratio, the specific surface of the particles that can be determined by the BET (Brunauer–Emmett–Teller) technique [24], which can be complemented with zero-potential measurements. This parameter influences the chemical reactivity and the flowability of the particles.

**Figure 4:**
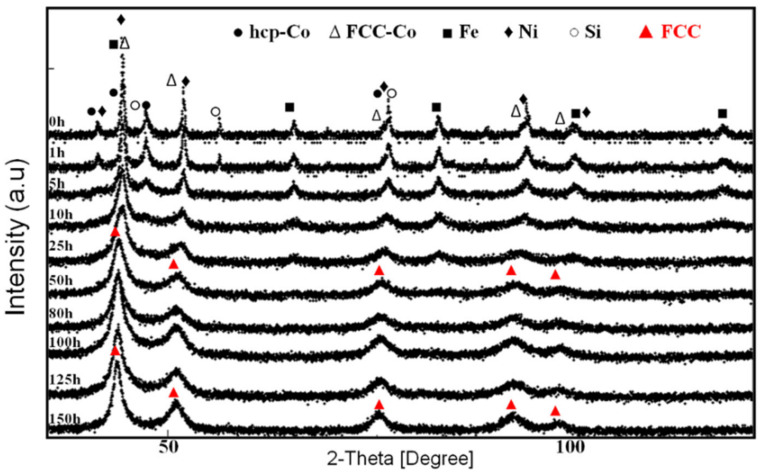
X-ray diffraction (XRD) patterns of FeCoNiB_0.5_Si_0.5_ powders as a function of mechanical alloying time [25].

The most common technique to detect the formation of a single solid solution in a single crystallographic phase is X-ray diffraction (XRD). At short milling times, the coexistence of several solid solutions is detected, the formation of which is influenced by the different diffusivities and the atomic percentage of each element. As the milling time increases, the tendency is for a single phase to form. Figure 4 shows the diffractograms of a high-entropy Fe-Co-Ni-Si-B alloy at various milling times [25]. In this paper, the final solid solution is an FCC phase evolving from the initial Ni FCC phase.

One of the characteristics of mechanical alloying is the formation of nanostructured materials [26]. There are several techniques to obtain nanomaterials, such as severe plastic deformation, physical vapor deposition, or mechanical alloying [27]. Concerning mechanical alloying, one example is the production of nanocrystalline equiatomic AlCoFeCrVNi and AlCoFeCrVTi high-entropy alloys, which were successfully synthesized by MA of the elemental powders followed by pressure sintering [28]. The milling procedure provokes solid-solution hardening and strong lattice distortion, which enhances the high-strength behavior of AlCoFeCrVNi and AlCoFeCrVTi. These phenomena are favored by several factors: (a) the diffusion during milling, (b) the lattice strengthening (due to the different atomic radii of the substitutional elements in the crystallographic phase), (c) the nanostructured state, (d) the grain boundary strengthening (due to the high percentage of atoms in the grain boundaries), and (e) the second-phase strengthening (due to the addition or the precipitation of secondary phases).

Likewise, mechanical alloying provokes a high density of crystallographic defects that can produce the formation of an amorphous phase [29]. The main mechanisms are the accumulation of defects (dislocation-mediated elastic instability) and the strain-induced amorphization (depressed dislocation nucleation favored by inhomogeneous atomic environments).

### 2.2. Consolidation and Additive Manufacturing

The specimens produced by mechanical alloying are powders. Thus, to obtain bulk specimens, further consolidation is needed. During the consolidation process, the specimens tend to shed the highly stored energy due to the milling and reach a lower-energy equilibrium configuration.

**Figure 5:**
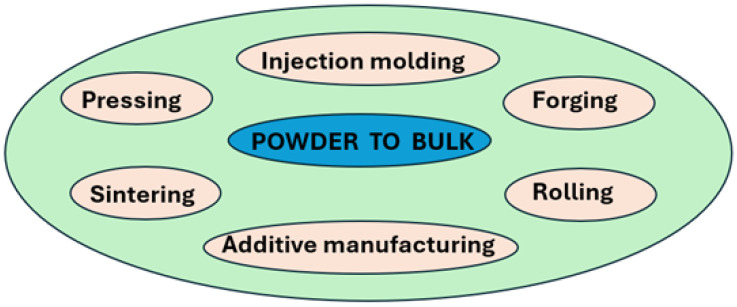
Schema of the pathways to produce bulk specimens with the mechanically alloyed powdered alloys.

**Figure 6:**
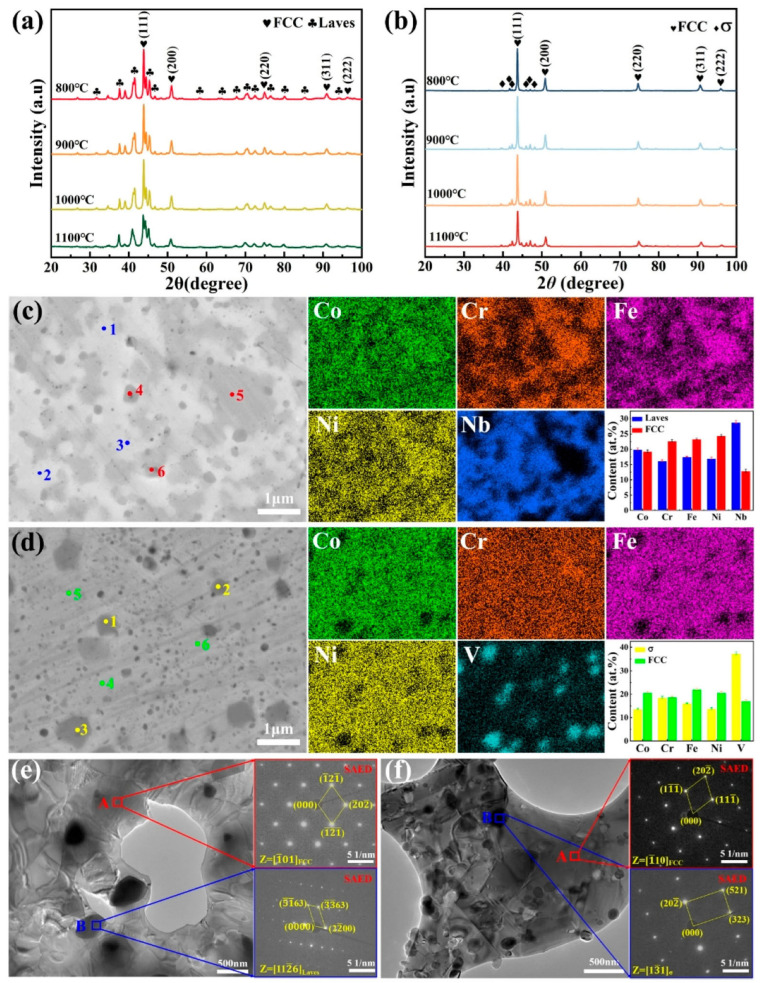
XRD patterns, SEM images, EDS elemental maps, and TEM images of sintered CoCrFeNiNb (**a**,**c**,**e**) and CoCrFeNiV (**b**,**d**,**f**) HEAs at different temperatures. (**a**,**b**) XRD patterns from 800 °C to 1100 °C; (**c**,**d**) elemental distributions and (**e**,**f**) TEM and corresponding SAED patterns of samples sintered at 1100 °C. The marked points 1, 2, and 3 correspond to the Laves phase and σ-phase regions, while points 4, 5, and 6 represent the FCC phase regions (**c**,**d**). The compositional statistics of these different regions are displayed in the corresponding bar charts [31].

Figure 5 is a schema of several procedures applied to obtain bulk parts/pieces: pressing, sintering, forging, rolling, injection molding, and, recently, additive manufacturing (AM). The methods included in Figure 5 are a classification of different pathways to produce bulk specimens. Pressing includes uniaxial and isostatic pressing, cold, warm, or hot pressing, and extrusion. They can be combined, such as pressing and further sintering. Moreover, controlled annealing for debinding, microstructure optimization, or porosity control is sometimes applied. Concerning sintering, spark plasma sintering (SPS), vacuum hot press sintering (VHPS), and microwave-assisted sintering (MAS) are three of the most important HEA consolidation technologies [30]. The main technological issue of sintering processes is the control of the final microstructure. Figure 6 shows CoCrFeNiNb sintered HEA XRD patterns, SEM images, EDS elemental maps, and TEM images [31]. It is an example of the formation of several phases during the sintering process, affecting the microstructure and the mechanical properties.

The cold pressing followed by sintering produces specimens with a density around 90% that of the theoretical value. Thus, a brittle fracture is possible. The sintering also favors the crystalline grain growth. The hot isostatic pressing facilitates higher density (about 98%) and can induce plasticity. The spark plasma sintering densities are above 99% and can retain the nanocrystallinity. The high-pressure torsion near the 100% density results in a high defect density and high hardness. The consolidation parameters are chosen to improve the density and reduce the porosity. An increase in relative density is found by increasing the pressure, the temperature, or the time in conventional consolidation methods. Nevertheless, an increase in pressure and temperature can favor the formation of undesired phases, the loss of nanocrystallinity, or the formation of cracks. Regarding the additive manufacturing methods, one of the main parameters is usually associated with energy transfer. In techniques such as laser bed powder fusion, there is also the volumetric energy density (VED). A low VED leads to lack-of-fusion defects and reduced relative density, whereas an excessive VED may induce cracks, the formation of metastable phases and pores formation. Thus, the effect of the energy density on specimen density is as follows: (a) at low VED, lack of fusion and high porosity, (b) at intermediate VED, a maximum densification, and (c) at excessive VED, keyhole pores and evaporation with a density drop.

There are general trends; specific examples are given in Table 4.

For additive manufacturing techniques, sometimes, the powder’s shape is previously modified by laser spheroidization [38]. The spherical shape and the control of the particle size distribution influence the flowability during the AM process.

**Figure 7:**
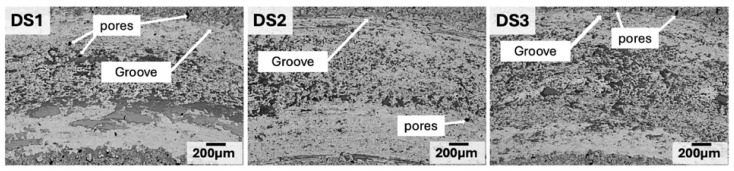
SEM of the worn surfaces of the HEA AlCoCrFeNi after dry sliding wear testing [40].

One specific case is the production of coatings with the MA powders. High-entropy alloy powders synthesized by mechanical alloying were used as feedstock to prepare coatings. In the formation of coatings, two aspects to take into account are the adhesion between the coating material and the substrate material and the homogeneity (thickness, microstructure) of the coating itself. Regarding the powders, the mechanically alloyed powder should have an appropriate shape, particle size distribution, flowability, cross-sectional microstructure, and phase structure [39]. Thus, to check the particle size distribution and shape of the particles produced under different milling conditions, it is suggested that the material to be used as feedstock be selected. Techniques widely applied are based on spraying, deposition, sputtering, or electrodeposition. Figure 7 shows SEM images of HEA AlCoCrFeNi coatings after dry sliding wear testing [40].

In a previous study [41], the authors studied FeCoNiCrAl and FeCoNiCrMn coatings by atmospheric plasma spraying. Typical analyses were the hardness, the friction coefficient, wear resistance, wear fatigue, sliding wear, and corrosion resistance. In the case of electrodeposition, the surfactants play a critical role in the electrodeposition of composite coatings because they modify the surface charge of the reinforcement particles. Thus, an improvement in the wettability of the particles is found. This phenomenon favors the agglomeration of the particles (by favoring the homogeneous dispersion).

HEA can be used as reinforcement particles. Ventakesh et al. [42] introduce milled CoCrMoNiW HEA reinforcement particles to electrodeposited Ni-HEA coatings. In this work, the BCC structure of the HEA particles and the minor FCC phase in the particles improve the compatibility between the Ni matrix and the HEA reinforcement particles.

An alternative to producing HEA bulk specimens is composite production. High entropy alloy composites (HEACs) are a new class of metal matrix composites involving a second phase, such as carbides, borides, and nitrides, in high-entropy alloy matrices. The percentage of the reinforcement ranges between 5% and 30%. As mentioned previously, the different sizes of the atoms of the HEA cause a high lattice distortion of the crystalline structure. This distortion acts as a barrier to the dislocation motion. As a consequence, it produces the strengthening of the matrix. Likewise, this phenomenon improves the strength–ductility synergistic combination. Furthermore, the final microstructure is usually modified by the consolidation method.

An example of HEAC composites is the reinforcement with carbides. Fe_18_Ni_23_Co_25_Cr_21_Mo_8_WNb_3_C_2_ was prepared by mechanical alloying and hot-pressing sintering. The sintered HEA composite contains the matrix and a second phase (M_6_C carbide) [43]. The HEAs can be mixed with specific elements to improve their properties. A TiCrZrFeNi HEA was mixed with Mg to produce a composite [44].

## 3. Properties

A typical classification of the properties is to differentiate the mechanical properties (including the wear resistance) from the corrosion resistance and functional properties (magnetic, electrical, optical, hydrogen storage, radiation shielding, and so on).

### 3.1. Mechanical Behavior

The most analyzed mechanical properties of the HEAs are the yield strength, the ductility, the hardness, the wear resistance, and the fatigue. Thus, the main desired mechanical properties of HEAs are elastic modulus, high strength, high hardness, wear resistance, and high toughness [45].

The increase in the milling energy provokes severe plastic deformation, critical defect density, grain refinement, and phase metastability. These features enhanced the mechanical properties [46,47]. The milling energy is primarily stored in the material through the generation of lattice defects. The as-milled powders are subjected to continuous fracture and cold welding mainly due to the collisions in the milling media (balls, containers). The main effects are outlined below [48]:

(A) Dislocation accumulation: Dislocation density increases usually exponentially until a maximum value, reaching levels as high as 10^15^ to 10^16^ m^−2^.
(B) Vacancy generation: The high strain rate produces the supersaturation of vacancies, which enhances the atomic diffusion (even at low and medium temperatures).
(C) Energy storage: The Gibbs free energy of the system provides the driving force for phase transformations that were unexpected under equilibrium conditions.

With respect to the reduction in the grain size and the grain refinement, the general trend is to achieve a minimum grain size. The main features are outlined below:

(A) Grain refinement: A high density of dislocations favors the formation of sub-grains. These sub-grains are free to rotate and to evolve into high-angle grain boundaries (nanocrystals).
(B) Grain size balance: The final grain size is a competitive balance between the grain refinement (driven by milling energy) and the recovery and recrystallization (driven by the heat generated during impacts). The grain refinement is driven by the milling energy, whereas the recovery and recrystallization are driven by the heat generated during the milling procedure (collisions, abrasion).
(C) Grain size limit: It is generally linked to several properties of the powdered material, such as the melting temperature and the stacking fault energy. For example, harder materials with high melting points typically achieve a smaller minimum grain size.

The formation of metastable phases, out of the equilibrium phase diagram, leads to the formation of the following:

(A) Supersaturated solid solutions: forcing elements with zero solubility (like Cu and Fe) to mix at the atomic level.
(B) Amorphous phase: If the defect density rises so high that the long-range crystalline order is lost, the energy provokes the evolution of the lattice to an amorphous (non-crystalline) state.
(C) Intermediate phase: In HEAs, metastable intermediate phases will be formed at low milling times.
(D) Allotropic transitions: The impacts during milling provoke a local high pressure, favoring the transition to high-pressure phases.

Table 5 shows the effect of the milling energy on several mechanical properties, remarking the main mechanisms involved.

With respect to the hardness, there are several mechanisms favoring mechanical hardening and strengthening: solid solution, strain, precipitation, and grain boundary strengthening. The hardening is detected due to the HEA’s formation of a solid solution with a high density of crystallographic defects (due to the mechanical alloying process) and a reduced crystallite size (a low crystallite size), implying a high density of crystallographic defects. The yield strength of an HEA can be expressed as the summation of every individual contribution (internal, solid solution, precipitation, grain boundary, dislocation).

The mechanical energy input and the rate of work hardening of the material influence the rate of structural refinement in HEAs. Thus, the increase in the milling time is a key factor, having a positive effect on the resulting mechanical properties of HEAs [51].

The crystallite size affects the mechanical response. The grain refinement provoked by MA produces nanocrystalline phases (with a crystallite size in the range between 10 and 30 nm). The smallest grains are usually linked to an increase in strength. Nevertheless, in MA, softening has been found at minute crystalline sizes, which is probably due to a diminution of the dislocation-induced plasticity by the increasing of grain boundary sliding. Likewise, as the milling time increases, the hardness also increases at low–medium milling times. At higher milling times, when the equilibrium between fracture and cold welding reaches an equilibrium, the hardness remains stable. Concerning the influence of the milling time on the mechanical response, other effects appear with the milling time increase in addition to the increase in yield strength and hardness. One is that the brittleness increases due to the contamination that can induce Orowan strengthening. A second effect is that the associated increase in chemical homogeneity tends to stabilize the mechanical response. A third effect is the induced disordering, favoring the formation of metastable phases, such as amorphous phases, with a different mechanical response. Table 6 shows a comparison of the strengthening mechanisms in MA processing and the magnitude usually achieved in HEAs.

One of the main issues influencing the mechanical response is the crystalline phase formed. HEA’s criteria are used to determine the expected crystallographic phase. Nevertheless, MA can induce the formation of a metastable phase. If an FCC phase is formed, it is expected for there to be high ductility and a relatively low yield strength. If a BCC is formed, it has high strength and probably room-temperature brittleness. If both phases (BCC and FCC) coexist or precipitates are formed, induced plasticity is expected.

Microstructural changes are induced by annealing. If a secondary phase is formed, there is additional dispersion strengthening. Thus, the total strength increases due to the combined effects of the nanocrystalline and secondary phases’ strength.

**Figure 8:**
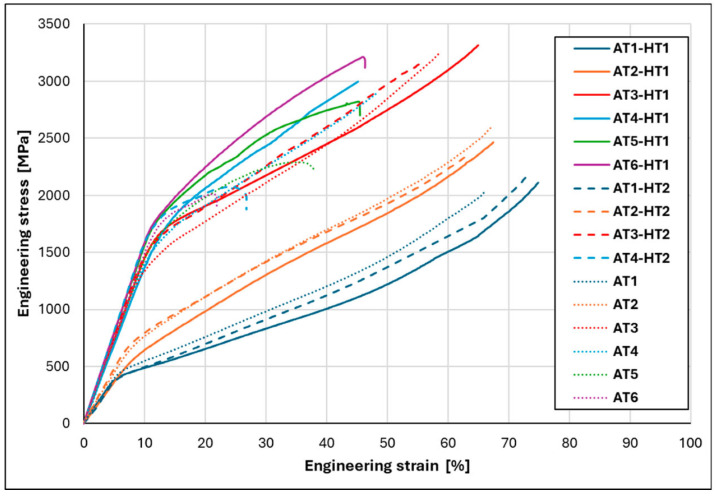
Comparison of compressive engineering stress–strain curves of Al_x_Ti_x_CrFe_2_Ni alloys after homogenization annealing under HT1 and HT2 conditions [52].

One of the most typical mechanical experiments is the stress–strain test. It provides information on both elastic and plastic behavior when present. Figure 8 shows several engineering stress–strain curves of Al-Ti-Cr-Fe-Ni bulk HEAs [52].

Wear resistance is related to the hardening. It is a key factor for HEAs in so many engineering fields, such as on marine parts, including drill pipe bits and propellers. The hardness of the worn surface increases due to the highly mechanically deformed grains and the high density of dislocations [53]. Furthermore, it is known that high stress favors fracture due to the crack initiation.

**Figure 9:**
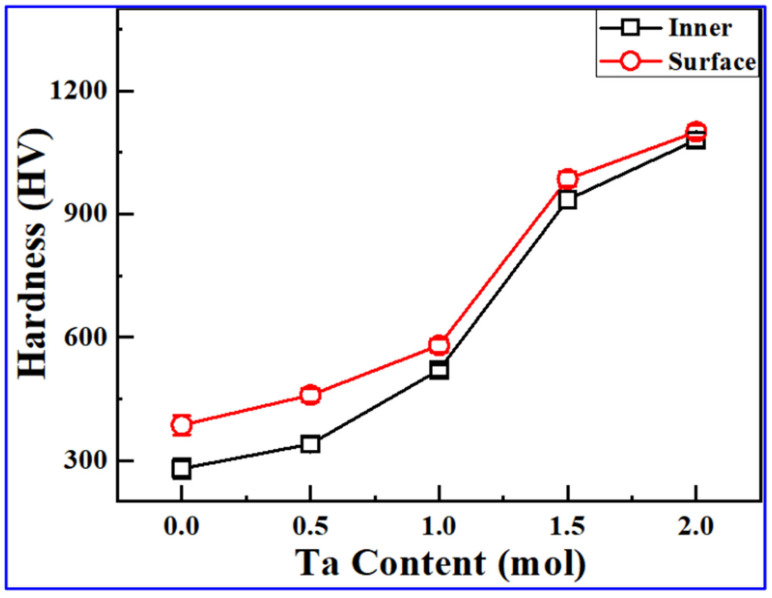
Surface and inner hardness of CoCrFeNiTax bulk HEAs [54].

The hardness can be modified with the milling time or by composition. For example, it has been found that upon increasing the Ta content, the alloy transformed into an “FCC + hexagonal close-packed (HCP) Laves phase in a dual-phase system [54], as shown in Figure 9.

The hardness is influenced by the milling time because MA induces severe plastic deformation. A high density of defects and the grain refinement enhance the hardness. As the milling time increases, the tendency is to form a solid-solution with high hardness and strength. A rapid increase in the hardness is found at low milling times (until 10–20 h) due to Hall–Petch effect. At intermediate and long milling times, the hardness tends to stabilize. Table 7 shows several quantitative results from HEA systems.

The grain size reduction is also enhanced by an optimized ball-to-powder weight ratio. Thus, a higher BPR increases the collision probability, the accumulation of dislocations, and a dynamic recovery, achieving a quick grain size reduction.

Table 8 shows the crystallite size of several milled HEAs. As a first approach, it is considered that the relationship between grain size and milling energy (driven by BPR) follows a power-law decay (with a theoretical limit). The nanocrystalline size is that of the main phase, because in some cases, in HEAs produced by MA, more than one phase remains.

**Figure 10:**
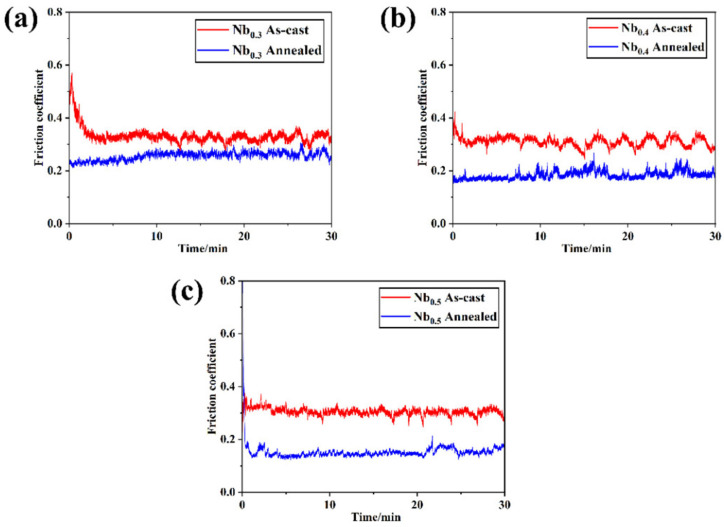
Friction coefficient of HEAs at different contents of Nb: (**a**) Nb_0.3_, (**b**) Nb_0.4_, and (**c**) Nb_0.5_ [65].

Wear resistance and friction were also tested. The precipitation strengthening after the annealing treatment and the lubrication effect of the FCC phase are the mechanisms for the significant improvement in wear resistance. The morphology of the samples indicates that the wear mechanism of the alloy includes adhesive wear, abrasive wear, and a certain degree of oxidation wear [65]. Figure 10 shows the evolution of friction coefficients with time of HEAs at different contents of Nb.

**Figure 11:**
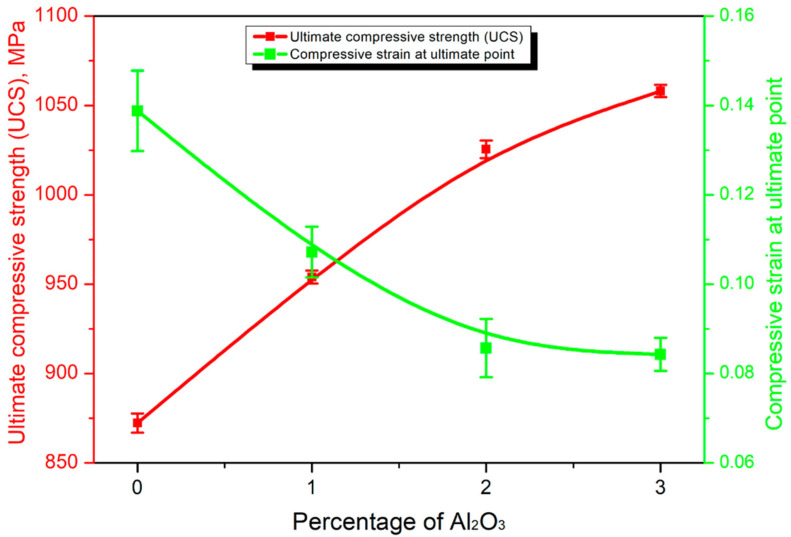
Variation in the ultimate compressive strength (UCS) and compressive ultimate point with the function of the Al_2_O_3_ in HEA composites [66].

Other mechanical properties are strength and toughness. With respect to strength, Shivasaskaran et al. detected that the increase in the Al_2_O_3_ content provokes a gradual increase in the hardness and mechanical strength, as shown in Figure 11 [66].

Concerning the toughness, the increase in the strength is associated with a decrease in the toughness. Nevertheless, the ultra-fine crystallites formed by milling improve strength without a drastic loss in toughness [67].

Obviously, the mechanical properties are influenced by the porosity. The ideal consolidated specimens should reach 100% of full density. Nevertheless, the processing parameters can induce cracks and/or pore formation. Higher relative density enhances the mechanical properties and helps to prevent the fracture of the bulk specimens.

The mechanical properties of the specimens consolidated from mechanically alloyed powders differ from those of cast alloys and alloys produced by additive manufacturing. The mechanically alloyed samples consolidated by traditional techniques such as spark plasma sintering usually have the highest hardness and yield strength due to nanocrystalline grain sizes, whereas the casting procedure due to the coarse grains often provokes a lower strength and higher ductility. In the last few decades, additive manufacturing methods have been applied. The AM tendency is to produce intermediate properties. It is the consequence of a balance between the refined microstructures and the high density. Table 9 shows different mechanical properties of bulk specimens produced by consolidating milled HEAs or after production by casting methods or additive manufacturing.

### 3.2. Chemical Properties

Corrosion resistance (including oxidation at high temperature) is considered a chemical property. As remarked in a previous section, MA favors the surface oxidation of the ball-milled powders due to the increase in the surface/volume ratio.

The typical analyses are compositional, electrochemical, or spectroscopic. It is known that the solid-solution microstructure of the HEAs facilitates the application as new catalyst materials for CO oxidation, ammonia oxidation, oxygen reduction, and so on [68]. Likewise, non-stoichiometric HEAs permit customizing the composition. These specimens can be applied as electrochemical sensors [69].

**Figure 12:**
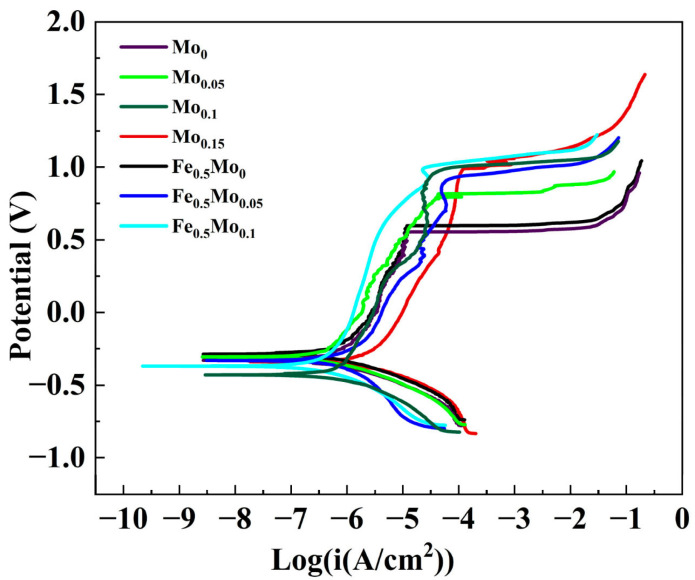
The polarization curves of CoCrNiFeyMox HEAs in a 60 °C, 3.5 wt.% NaCl solution [73].

The development of HEAs for electrocatalytic applications has some advantages with respect to other production techniques: chemical co-reduction, solution combustion, or sol–gel. Mechanically alloyed materials are solvent-free and allow controlled bulk production, avoiding high energy consumption; they help the development of materials with thermodynamic immiscibility; and they hinder elemental segregation [70]. Studies have been performed on FeCoNiCrP [71], FeCoNiMo(Cr,Cu) [72], and CoCrNiFeMo [73]. Figure 12 shows the polarization curves of CoCrNiFe_y_Mo_x_ HEAs.

**Figure 13:**
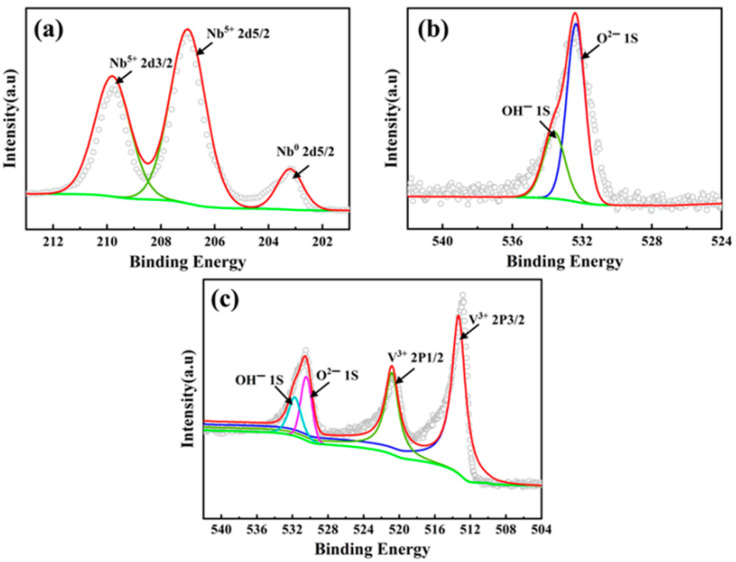
XPS spectra of the surface oxide layers formed on CoCrFeNiNb and CoCrFeNiV HEAs after electrochemical corrosion in a 3.5 wt.% NaCl solution. (**a**) Nb 2d spectrum of CoCrFeNiNb HEA, (**b**) O 1s spectrum of CoCrFeNiNb HEA, (**c**) V 2p and O 1s spectrums of CoCrFeNiV HEA. Gray circles represent the experimental data, and red curves show the fitting results [31]. Baseline: green line. Red line: Sum of the fitted sub-spectra.

The electrochemical corrosion behavior is also analyzed with X-ray photoelectron spectroscopy (XPS), which analyzes the elements constituting the sample surface and depth profile, its composition, and the chemical bonding state. Figure 13 shows the XPS spectra of the surface oxide layers on CoCrFeNiNb and CoCrFeNiV HEAs.

Some research works are focused on a specific element, such as Cu. Asl et al. [74] determined that the addition of Cu can provoke a loss of corrosion resistance due to the combination of pitting corrosion and micro-galvanic cells.

### 3.3. Functional Properties and Applications

Several studies are focused on the functional properties of the HEA alloys [68]. They are related to specific applications. In vitro corrosion is a property that can be considered functional due to its applicability in biomedicine. The magnetocaloric HEAs are magnetic material candidates for application in magnetic refrigeration. The radiation absorption materials are useful in health, communications, and military applications. Thus, the following classification is one of the possible ways to classify HEAs.

#### 3.3.1. Health

Regarding biomedicine, HEA materials are checked as candidates for in vitro corrosion prevention [75]. The high-entropy effect stabilizes the corrosion-resistant phase in highly aggressive media. The mixing of biocompatible elements, such as Ti, Al, Nb, Zr, Mo, Ag, and Ta, is an option. Likewise, some of these elements can replace Al in Ti-Al-based alloys. Results indicate that the in vitro corrosion protection of the bulk samples (sintered from powders) is enhanced by increasing the heating rate.

**Figure 14:**
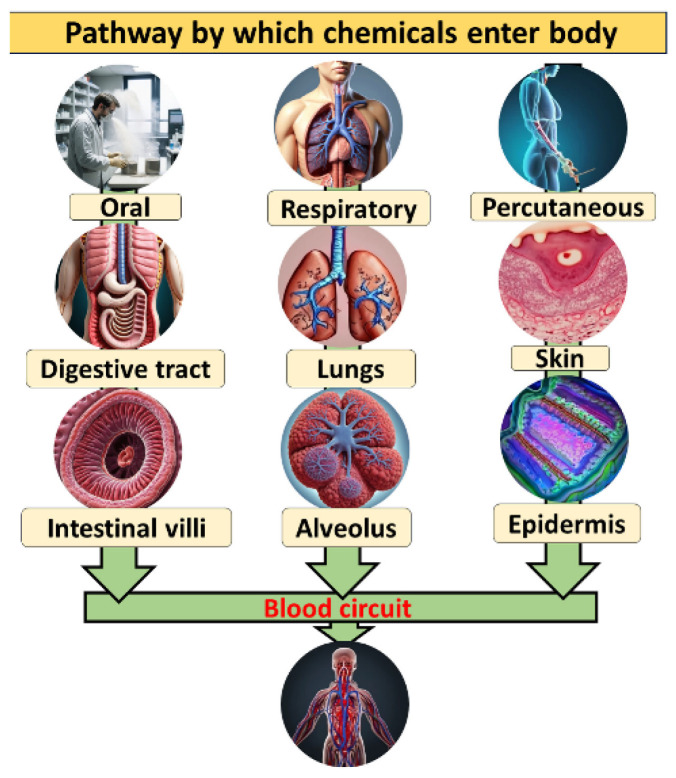
Schematic presentation of the pathway by which chemicals enter the body [76].

Some studies also analyzed the antibacterial behavior [74]. In this study, the HEA bulk specimens exhibited enhanced antibacterial activities against both *E. coli* and *S. aureus* bacteria. Figure 14 shows a schema of the HEA health hazards [76]. It is recommended to avoid reactive metals, toxic elements, and rare-earth elements. The reactive metals are very active in chemical reactions with oxygen and moisture due to their low electronegativity. This reactive nature could result in the formation of oxides. The main problems associated with the toxic elements are due to their chemical reactivity, solubility, electronegativity, oxidation states, and radioactivity, affecting DNA stability, biological processes, and physiological functions. The rare-earth elements also have traces of radioactive elements and are reactive, are toxic, and can contaminate the environment.

#### 3.3.2. Refractory Radiation

Also related to health is the problem associated with the absorption of radiation, whether in microwave or radio frequencies. It is necessary to develop new materials that can absorb at least 90–95% of radiation to minimize the energy–matter interaction with human beings. These materials need lightweight behavior and environmental stability. These materials also have military and aerospace applications and telecommunications applications, as they allow for radiation modulation. The exponential growth of the information and communication networks has provoked an increase in these, which are harmful to living beings. With respect to MA, this technique favors the formation of amorphous/crystalline mixtures and the use of surfactant-influenced morphology. It is known that flake morphology enhances microwave scattering cross-sections and that the solid-solution crystalline microstructures with defects also enhance broadband absorption [77]. Regarding the composition, there are Fe-Co-Ni-based elements with the addition of Cr, Cu, Mn, Ti, Al, B, Mn, or V [78]. It is known that irradiation can induce elemental activation [79].

The radiation shielding also includes other electromagnetic radiations, such as gamma-ray attenuation, and particle radiation, such as neutron radiation. The key irradiation parameters are the type of irradiation (neutrons, heavy ions, electrons, or gamma rays); the particle energy (higher value implies higher depth of penetration and damage); the flux (number of particles per unit area and unit time); and the fluence (total particles per unit area), determining the total dose, the irradiation temperature, or the linear energy transfer associated to the radiation quality. A review of the applications of irradiated HEAs is given in ref. [80].

**Figure 15:**
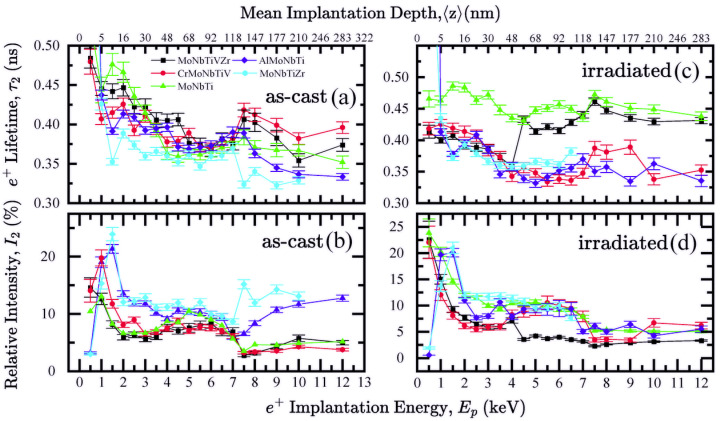
Lifetimes for (**a**) as-cast and (**c**) irradiated samples and their intensities (**b**) and (**d**), respectively. In practice, about 2 or 3 lifetime components are measurable; however, the intensity components of base + Al and base + Zr appear to be weak but are non-zero [82].

Fe-Co-Ni-based HEA ferromagnetic alloys are an alternative to B_4_C and graphite due to the fast neutron removal capability [81]. The typical radiation of nuclear installations is necessary to ensure the structural integrity and longevity of the nuclear reactor parts. There are extreme conditions, and these materials should also have high thermal stability, corrosion resistance, and superior mechanical strength. The combination of these properties at high temperatures justifies the classification as refractory materials, as they are optimized for extreme conditions. Figure 15 shows lifetime and relative intensity as a function of the mean implantation depth and the implantation energy of Mo-Nb-Ti-based alloys [82]. In this study, the samples were produced by spark plasma sintering. The irradiation was conducted in a 3 MV tandem accelerator. Si ions were used to induce damage with a fluence of 1.43 × 10^15^ ions/cm^2^ at 10 MeV to avoid introducing Si impurities into the lattice.

The corrosion resistance is also compositionally dependent. Likewise, the minor addition of elements such as C, Al, Ti, or Be enhances the resistance.

The irradiation can induce structural changes in the microstructure of the HEAs:

(a) Segregation: The main mechanisms are the vacancy and the interstitial atomic ones.
(b) Dislocation: increasing the density of dislocations and the dislocation loops.
(c) Cavities: nucleation and growth of cavities. Bubbles: helium bubbles were provoked.
(d) Impurities: embrittlement from H, He, and impurities and H and He.
(e) Solubility: higher solubility by interstitial elements.

The impurities and the induced crystallographic defects can provoke hardening. Thus, the control of the composition and microstructure is necessary to optimize the corrosion resistance and further thermal stability for applications [83]. It is known that the low-activation elements required in nuclear applications tend to form BCC crystallographic solid solutions [84].

The induced irradiation damage also depends on the characteristics of the samples being irradiated: the atomic weight, bond strength, crystal structure, stacking fault energy, composition, and microstructure. Thus, the irradiation damage processes control the long-term stability of the materials’ response.

In aerospace, the shielding application is found in high-entropy carbides and related alloys due to their high density and radiation attenuation [85].

#### 3.3.3. Magnetic Applications

The Fe-Co-Ni-based alloys are candidates for applications for magnetic sensors and devices. Mn, Ti, Cr, Sn, V, Hf, Ga, or Al are commonly added to these alloys. There is an alternative to conventional Fe-rich and Co-rich soft magnetic alloys in high-frequency transformers, in relays, in magnetic shielding, in magnetic resonance images, and in magnetic switching. The reduction in the crystallite size facilitates magnetic softness. Nevertheless, the mechanically induced micro- and nanostrain provokes an increase in coercivity, an increase in magnetic hysteretic loss, and a reduction in the saturation magnetization. The high density of crystallographic defects makes the optimization of the magnetic order needed to obtain a high magnetization of saturation. The alternative is controlled annealing at low temperature to reduce the microstrain by thermal relaxation and avoid any crystallite size increase. The thermal relaxation facilitates the reduction in the coercivity and the soft magnetic behavior. Likewise, the addition of cobalt increases the magnetization of saturation, whereas the addition of nickel provokes its reduction. Concerning the working conditions, a key parameter is the Curie temperature of these alloys, which is a superior limit. The remanence and the squareness ratio of the as-milled powders have low values. The surface oxidation also provokes a drastic change in the magnetic behavior.

**Figure 16:**
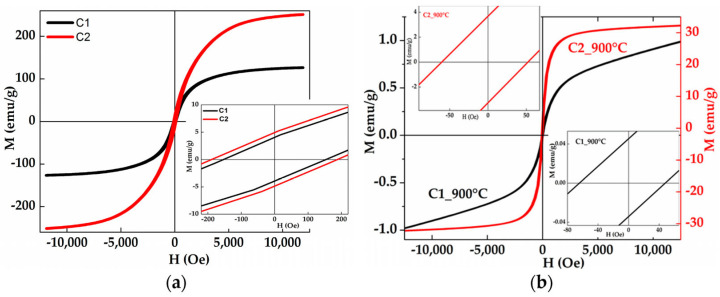
(**a**) Hysteresis loops of the mechanically alloyed Fe-Cr-Co-Ni-Mn HEAs C1 and C2 for 24 h; (**b**) C1_900 °C and C2_900 °C. Insets show the enlargement of the central parts [86].

The magnetic hysteresis loops (magnetization as a function of the external magnetic field) allow for the determination of the coercivity, the saturation magnetization, the remanence, and the squareness ratio. Figure 16 shows cycles of Fe-Cr-Co-Ni-Mn HEAs [86].

**Figure 17:**
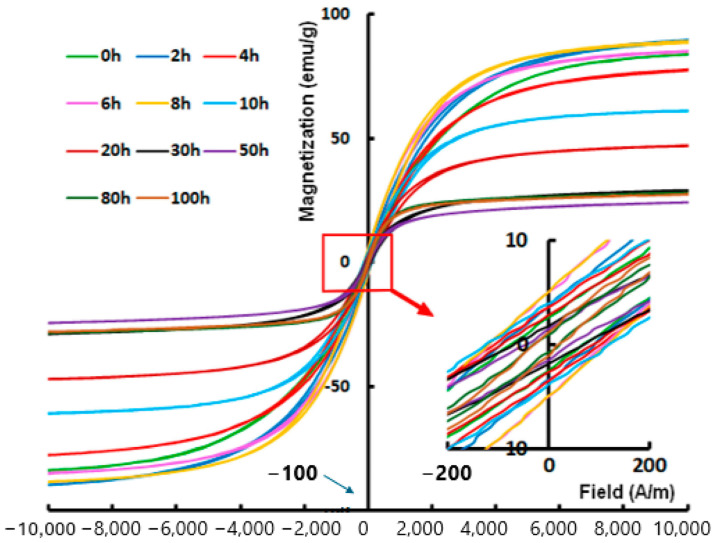
Typical hysteresis cycles (at 300 K) of the MA Fe_30_Co_20_Ni_20_Mn_20_Ti_10_ powder mixtures as a function of milling times [88].

The FCC and BCC phases produced by milling sometimes evolve to a unique FCC phase on sintering to produce bulk specimens [87]. Likewise, the phases evolve during milling, and consequently, the magnetic response. As they are multicomponent systems, the diffusion rates of the elements differ, affecting solid solution formation. Figure 17 shows magnetic results in an FeCoNiMnTi alloy at different milling times [88].

**Figure 18:**
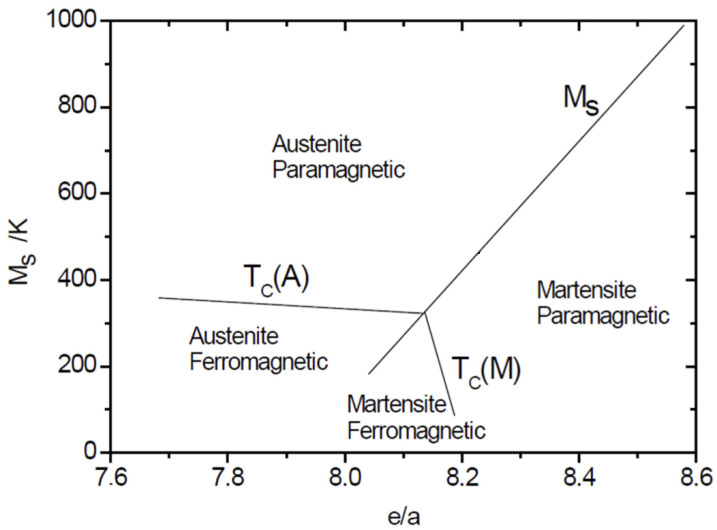
Martensitic start temperature versus average valence electron density, e/a [89].

Another family of soft magnetic materials is the magnetocaloric materials. These materials are characterized by high enthalpy and entropy change during the ferromagnetic-to-paramagnetic reversible transformation. It is the magnetocaloric effect. Some of these HEAs also have a structural transformation (hysteretic austenite to martensite transformation). Thus, shifting the composition drastically transforms the transition temperatures and the functional response. Figure 18 shows a diagram of the typical magnetostructural transformations [89].

These alloys are candidates for magnetic refrigeration devices. The main magnetocaloric parameters of the materials are the maximum magnetic entropy change, ΔS_M_, and the refrigerant capacity, RC. Concerning mechanical alloying, the produced powders have a high density of crystallographic defects, shifting and sometimes avoiding the transformations. The as-milled powders need to be annealed to recover the crystallographic structure. A milling process at low milling intensity and reduced milling time is recommended. Nevertheless, the crystallographic defects and the induced disorder make an ulterior customized annealing necessary to recover the crystallographic phase.

One of the magnetocaloric families is the (Mn, Fe)_2_(P, Si)-based alloys with the HCP-Fe_2_P-type phase. In this case, the magnetic-field-induced magneto-structural transformation consists of a transformation from the hexagonal to the orthorhombic phase. The large magnetization difference between the two phases favors the giant magnetocaloric effect [90].

As remarked in Table 1, many criteria have been applied in the analysis of HEAs’ behavior. One parameter introduced for magnetocaloric HEAs is the effective temperature, *T_eff_* [10]. This temperature is defined as the ratio between the increase in the metallic bonding energy of solid solutions with respect to segregated pure constituents and configurational entropy.

The surface oxidation layer inhibits the exchange coupling when two magnetic phases can interact, such as in soft-hard magnetic composites known as spring magnets.

#### 3.3.4. Energy

**Figure 19:**
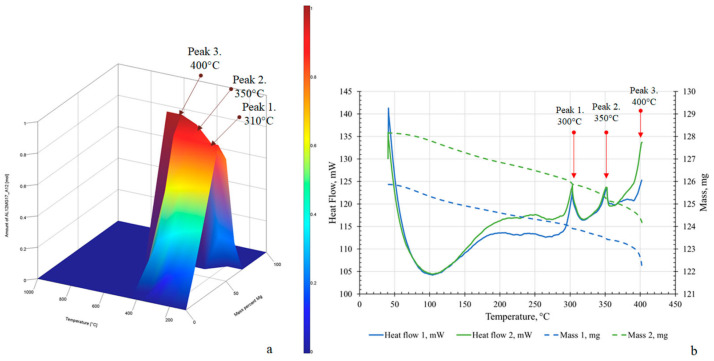
Thermodynamic modeling of heat capacity (**a**) and experimental TGA/DSC curves (**b**) for Mg_56_Al_44_ samples: green—after MS at BPR 20:1 followed by SPS at 350 °C and 20 min dispersion; blue—after MS at BPR 30:1 followed by SPS at 350 °C and 20 min dispersion [93].

Hydrogen storage is considered an energy vector. The hydrogen is stored in alloys and compounds, which are usually intermetallic compounds or Laves phases. MA favors the formation of supersaturated disordered solid solutions. Nevertheless, the crystallographic defects (vacancies and dislocations) act as trapping sites for hydrogen, modifying the kinetics and capacity of hydrogen storage. Likewise, it has been found that the presence of impurities can inhibit hydrogen diffusion by blocking interstitial sites [91]. Furthermore, it has been suggested, in Ti-V-Zr-Nb-Hf HEAs, that the size mismatch between the five elements improves the hydrogen content [92]. One of the traditional families is the Mn-Ni and Mn-Al-based alloys. Figure 19 shows the experimental calorimetry and thermogravimetry scans and the heat capacity of a Mn-Al alloy [93]. MA favors the nanocrystalline state, a high density of grain boundaries, and the hydrogen storage capacity.

**Figure 20:**
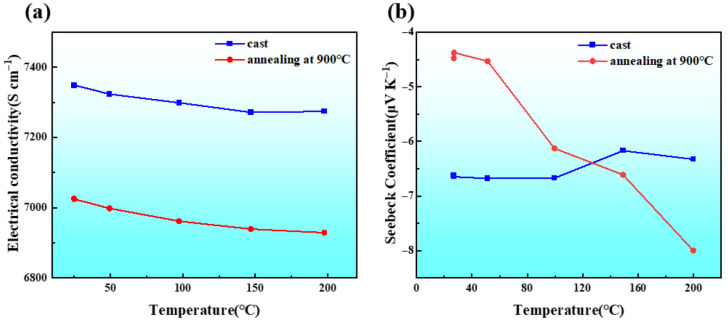
Variation in conductivity (**a**) and Seebeck coefficient (**b**) of Al_2_CoCrFeNi HEA with temperature [94].

Thermoelectric materials are of technological interest due to their applicability in energy generation and utilization systems. Regarding their properties, the main parameter is the figure of merit, ZT, which is related to properties such as the thermal conductivity, the electrical conductivity, and the Seebeck coefficient, with temperature being a determining factor. These materials have often been produced via mechanical alloying in powder form with the powder particles subsequently consolidated. Spark plasma sintering has been a widely used technique. The microstructural changes provoked by consolidation and sintering modify the alloys’ properties. Thus, sometimes, annealing is also performed. Figure 20 shows the electrical conductivity and the Seebeck coefficient evolution with the temperature (before and after annealing) of an Al_2_CoCrFeNi HEA [94]. MA-induced crystallographic defects diminish the electrical conductivity as conductive electrons easily interact with the atoms in the highly distorted lattice. Heat treatment can affect different parameters in various ways (increasing or decreasing); the desired combination is that it produces an increase in ZT.

Superconducting materials can also be produced by mechanical alloying. Their potential applications include energy production, efficient power devices, magnetic energy storage, and power transmission lines. Other energy materials are those related to photothermal conversion materials. There are so many promising functional properties of HEAs [68].

## 4. Recent Pathways

There are recent pathways associated with new families of high-entropy alloys. High-entropy oxides could be candidates for applications in electrochemistry, catalysis, energy storage, cutting-edge environmental protection, gas storage and sensing, electromagnetic wave absorption, shape-memory materials, and so on [95,96]. 

Concerning the control of functional properties, there are several issues. The well-designed thermal or thermo-mechanical treatment will affect the microstructure: solution, precipitates, twinning, and dislocations. Another strategy is the controlled addition of minor elements, which modify the chemical composition of the alloys. It is known that there is a close relationship between microstructure and functional response [97].

Current trends in production techniques revolve around the novelty of additive manufacturing techniques, AM, as an alternative to the traditional consolidation or sintering of HEAs. Currently, the predominant AM techniques utilized for HEAs include selective laser melting, selective laser sintering, powder bed fusion, direct-injection writing, binder jetting, extrusion, and photopolymerization [98,99]. Furthermore, some of these techniques also favor the formation of compositional gradient HEAs.

A technological challenge in additive manufacturing is the need for particles with a well-defined, spherical shape. Particle size can be controlled by sieving. However, particle sphericity cannot be achieved through mechanical alloying. The continuous fracturing and cold welding that occur during milling result in particles with a relatively high specific surface area. One option for achieving sphericity is a secondary processing step using laser spheroidization [38]. This secondary processing step allows for optimized feedstock. However, it can cause undesirable effects such as structural changes in the alloys and, in some cases, easy surface oxidation of the particles. For this, mechanical alloying has the competence of techniques such as gas atomization for the production of AM feedstocks [100].

Recently, machine learning (ML) has played an increasingly important role in the selection of composition and processing conditions and for customizing HEAs.

The machine learning models use all or part of the criteria introduced in Table 1. The feature-important analysis highlighted the necessity of optimal database information (composition, precursors, and final products’ microstructure and properties; processing conditions, including post-treatments; and so on). The main criteria found in the literature are the valence electron concentration and the melting temperature. Thus, both act as crucial factors in distinguishing the phase structures of HEAs because not all the parameters are given or calculated in the published information. In a recent work, seven of the parameters described in Table 1 were selected for machine learning analysis [100]. Each parameter influences a different issue, as shown in Table 10.

Prediction accuracy is one of the main factors to take into account in machine learning. It is recommended to be close to or higher than 90%. Thus, the enhancement in accuracy needs databases with high reliability. This high reliability should be based on well-executed and well-described (as many criteria as possible) scientific literature.

**Figure 21:**
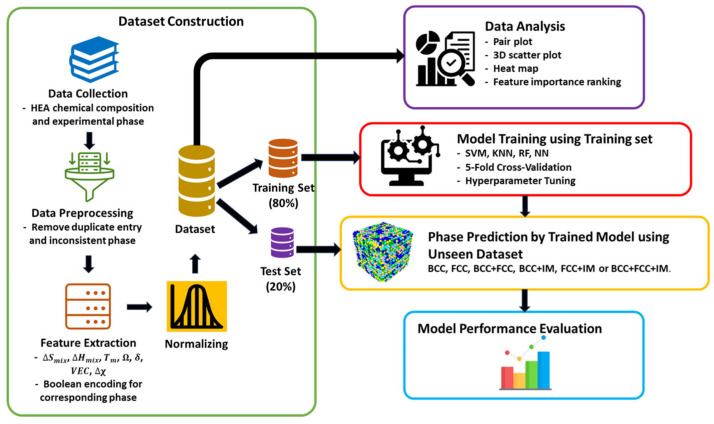
The comprehensive strategy for predicting the phases of high-entropy alloys [101].

Figure 21 shows a schema of the data analysis strategy performed in ref. [100] for predicting phase formation.

**Figure 22:**
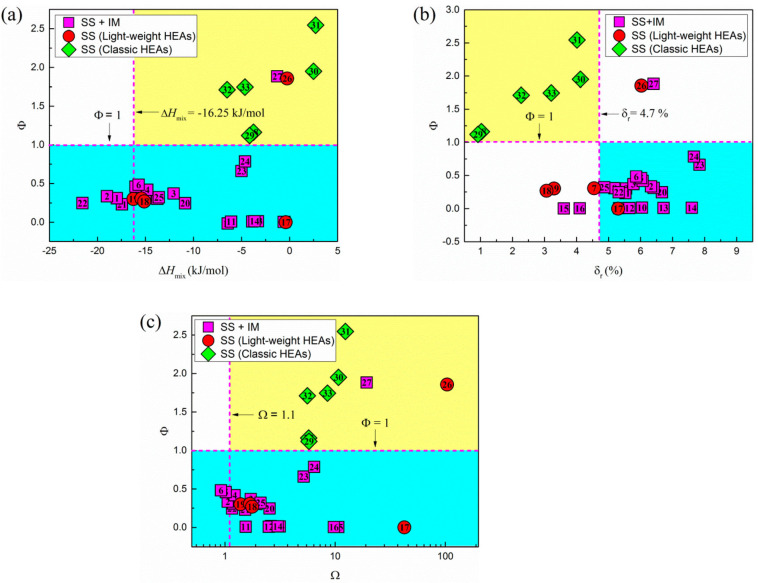
The variation in the thermodynamic parameter, ϕ, with respect to (**a**) $∆H_{mix}$, (**b**) δ and (**c**) Ω [102]. The dashed lines define different regions.

Machine learning permits establishing relationships and trends between the different HEA criteria. Feng et al. analyze the variation in several parameters with the ϕ parameter [102], as shown in Figure 22.

**Figure 23:**
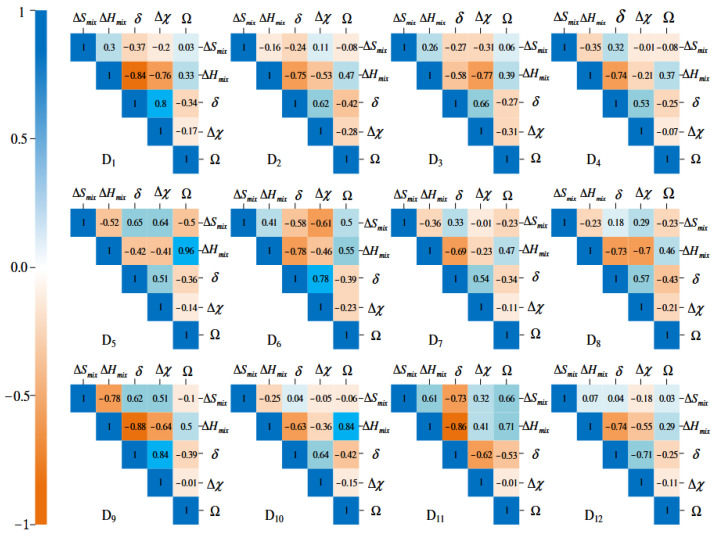
Heatmap of Pearson correlation coefficient matrix among 5 parameters in 12 datasets [103].

Hou et al. analyzed five parameters in twelve datasets [103]. The heatmap of the Pearson correlation coefficient matrix is given in Figure 23.

**Figure 24:**
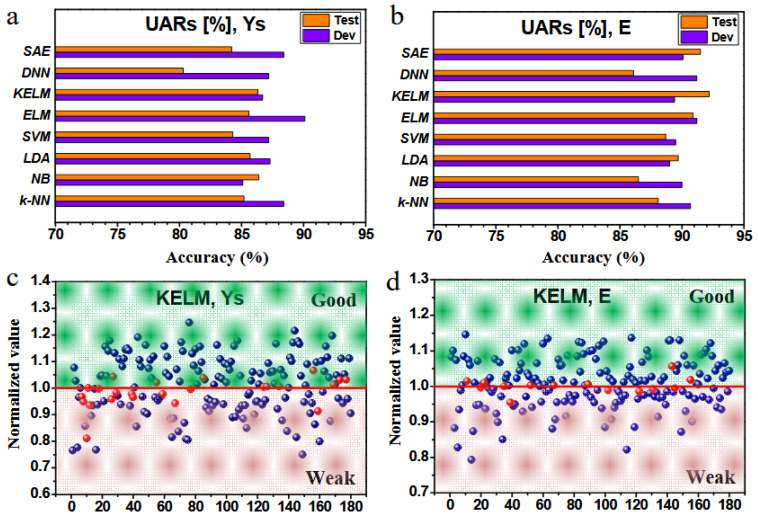
Results of ML model training and evaluation. The results (UARs in [%]) of eight ML models on the task of (**a**) yield stress and (**b**) Young’s modulus. The UARs shown for the dev set are the best UARs achieved by the optimal hyperparameters tuned for the corresponding model. The UARs shown for the test set are the final performance achieved by the model trained by the train plus dev sets within the optimal hyperparameters. Prediction results of the 180 non-equiatomic samples on the test set by the KELM model for (**c**) yield stress and (**d**) Young’s modulus. The red line is the benchmark line based on the value of the equiatomic sample; the results of non-equiatomic samples are normalized to the benchmark value, where the values above the line are classified to the ‘Good’ zone, and those below the line are classified to the ‘Weak’ zone. If the ML prediction matches the MD simulation, the point is colored blue, but otherwise, the point is colored red [104].

Practical applications of ML in HEAs are related to phase selection, stacking fault energy, strengthening mechanism, deformation mechanism, and mechanical properties. In atomistic simulation ML research, the authors analyzed results of models’ training and evaluation in several mechanical properties, as shown in Figure 24 [104].

**Figure 25:**
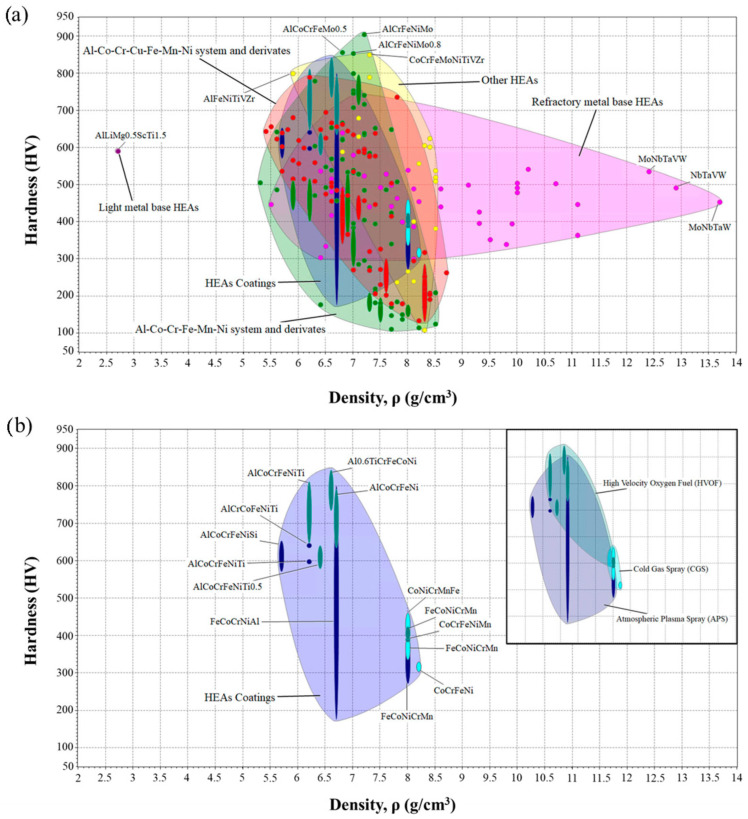
Hardness as a function of density for (**a**) the whole HEA database and (**b**) HEA coatings [105].

Regarding the HEAs database, there are some examples on the web. The High-Entropy Alloys Predicting Software (HEAPS, version 1.0) project is led by Pablo Martin St. Laurence, from the Polytechnic University of Catalonia (Spain), in collaboration with members of the Technical University Federico Santa María (Chile). Figure 25 shows two examples of maps built with this database [105].

The code version is also available for download at www.rpm.usm.cl and at https://github.com/pmartinsl/HEAPS, accessed on 20 January 2026. Another example of an application of this database can be seen in reference [12].

There are other databases, such as the Consolidated Database of High Entropy Materials (COD’HEM). This database consists of experimentally measured mechanical properties of high-entropy alloys (HEAs), which are found in COD’HEM by MCDC (https://codhem.cecas.clemson.edu/dashboard, accessed on 20 January 2026). Conventional software such as Thermo-Calc is introducing databases of HEAs (https://thermocalc.com/products/databases/high-entropy-alloys/, accessed on 20 January 2026).

In MA, there are specific processing parameters that will be taken into account to address optimized machine learning on the mechanically allowed samples such as the milling time, milling energy, or milling contamination. For microstructures, the development of metastable phases and the increasing in the crystallographic defects are taken into account.

Recently, artificial intelligence (AI) has also been applied to HEAs [106]. AI applications include the composition design, phase structure prediction, performance optimization, and new material discovery.

It is also recommended to integrate machine learning analysis with experimental and computational methods (ab initio and Monte Carlo) and the prospecting of the relationships between processing conditions, microstructures, and the mechanical and functional properties [107].

## 5. Conclusions

Mechanical alloying is one of the most widely used techniques for producing high-energy alloys (HEAs) in powder form. It facilitates the formation of metastable phases and a high density of crystallographic defects. Because it can be applied to a wide variety of alloys, it allows for the production of compounds for fields such as energy, sustainability, and healthcare. Optimizing the consolidation and sintering processes, as well as any subsequent heat or thermomechanical treatments, is necessary for the manufacturing of bulk components. The goal is for the final part to have an optimized microstructure for enhanced functional performance.

Among the new issues of research is the use of particles as feedstock in additive manufacturing processes, which necessitates controlling the particle size and morphology. Another area is the application of machine learning and additive manufacturing, typically in selecting the composition to optimize a specific functional property. In this case, generating comprehensive and rigorous databases is essential.

## Figures and Tables

**Table 1:** The criteria applied in scientific literature for HEA prediction.

Criteria	Equation	Units
Mixing entropy	$∆S_{mix}=-R\sum_{i=1}^{n}c_{i}\ln\left(c_{i}\right)$	J/(mol·K)
Standard deviation of mixing entropy	$\sigma_{∆S}=\sqrt{\sum_{i=1}^{n}{\left({∆S}_{i}-∆S_{mix}\right)}^{2}}$	J/(mol·K)
Mixing enthalpy	$∆H_{mix}=\sum_{i=1,i\neq j}^{n}4H_{ij}c_{i}c_{j}$	J/mol
Standard deviation of mixing enthalpy	$\sigma_{∆H}=\sqrt{\sum_{i=1,i\neq j}^{n}c_{i}c_{j}{\left(H_{ij}-∆H_{mix}\right)}^{2}}$	J/mol
Valence electron concentration	$VEC=\sum_{i=1}^{n}c_{i}VEC_{i}$	-
Standard deviation of valence electron concentration	$\sigma_{VEC}=\sqrt{\sum_{i=1}^{n}c_{i}{\left(VEC_{i}-VEC\right)}^{2}}$	-
Electronegativity	$\chi =\sum_{i=1}^{n}c_{i}\chi_{i}$	-
Electronegativity difference	$∆\chi =\sqrt{\sum_{i=1}^{n}c_{i}{\left(\chi_{i}-\chi \right)}^{2}}$	-
Average atomic radius	$a=\sum_{i=1}^{n}c_{i}r_{i}$	Å
Atomic size difference	$\delta =\sqrt{\sum_{i=1}^{n}c_{i}{\left(1-\frac{r_{i}}{a}\right)}^{2}}$	%
Melting temperature	$T_{m}=\sum_{i=1}^{n}c_{i}T_{i}$	K
Standard deviation of melting temperature	$\sigma_{T}=\sqrt{\sum_{i=1}^{n}c_{i}{\left(1-\frac{T_{i}}{T_{m}}\right)}^{2}}$	-
Effective temperature	$T_{eff}=\frac{∆U_{0}}{∆S_{mix}}$	K
Ω parameter	$\Omega =\frac{T_{m}∆S_{mix}}{\left|∆H_{mix}\right|}$	-
Φ parameter	$\phi \left(f\right)=\frac{∆S_{mix}-\frac{∆H_{mix}}{T_{m}}}{\left|S^{XS}(f)\right|}$	-
Λ parameter	$\Lambda =\frac{∆S_{mix}}{\delta_{r}^{2}}$	J/(mol K)

**Table 2:** Influence of several milling parameters on the microstructure and the effect on HEA specimens [4,18,19,20]. An upward arrow indicates the MA parameter increase.

MA Parameter	Microstructure	Effect on HEAs
↑ Milling Time	Reduction in grain size; increased homogeneity	Higher hardness and strength
↑ Milling Speed	Enhanced energy input; accelerated alloying	Faster phase stabilization
↑ BPR	Increased collision frequency; severe lattice distortion	Improved solid solubility
↑ PCA	Reduced cold welding; prevent agglomeration	Controlled particle size

**Table 3:** Features comparison between MA, cast, and additive manufacturing specimens.

Feature	Mechanical Alloying	Cast	AM
Processing State	Solid state	Liquid state	Liquid/solid
Crystallite Size	Nanocrystalline–amorphous	Coarse crystallites	Fine/columnar
Homogeneity	Good (long milling times)	Good (macroscopic level)	Variable
Contamination	High (milling tools, oxidation)	Very low	Low–medium

**Table 4:** Density values of consolidated ball-milled powders by applying different consolidation methods (traditional, additive manufacturing) in powdered HEAs: AlCrFeMnNiW [32], MgAlTiVFeCo [33], FeCoCrNiMnTiC [34], AlCoCrFeNi [35], CoCrFeMnNi [36], AlCoCrFeNiTi [37]. VED: volumetric energy density.

HEA Alloy	Consolidation Technique	Consolidation Parameters	Density (%)
AlCrFeMnNiW	Spark plasma sintering	900 °C/50 MPa/5 min	83%
MgAlTiVFeCo	Cold press + sintering	850–1000 °C/25 MPa/20 min	85.8–96.7%
FeCoCrNiMnTiC	Vacuum hot pressing	850–1000 °C/50 MPa/1 h	98–100%
AlCoCrFeNi	Hot pressing	1100 °C/30 MPa/2 h	99–100%
CoCrFeMnNi	Laser powder bed fusion	VED ≈ 104 J/mm^3^	96.3–99.4%
AlCoCrFeNiTi	Direct energy deposition	Laser power: 500–800 W	95–97%

**Table 5:** Effect of the milling energy on several mechanical properties and mechanisms involved [49,50].

Property	MA Energy Effect	Mechanism
Yield Strength	Significant Increase	Hall–Petch strengthening: 1/d effect from nanograins.
Hardness	Increase	Strain hardening and grain boundary impediment of dislocations.
Ductility	Decrease	Limited dislocation storage capacity in very small grains.
Fatigue Life	Variable	Improved by refined microstructure but hindered by any residual porosity.

**Table 6:** Strengthening mechanism, the relation with the MA processing, and the effect in HEAs.

Mechanism	MA Process	HEAs Effect
Solid solution	Multi-element mixing	Very high
Hall–Petch	Severe plastic deformation	High
Dislocation	Mechanical impact	Moderate to high
Precipitation	Contamination/PCA	Variable

**Table 7:** Milling time, crystallite size, hardness, and phase evolution of several HEA systems: AlCoCrCuFeNi [55], CoCrFeNiMn [56], AlFeNiCoMn [57], AlCoCuNiTi [58], and TiTaVWCr [59].

HEA Alloy	Milling Time (h)	Hardness (Initial-Final) HV	Hardening Mechanism
AlCoCrCuFeNI	50	400 HV → 850 HV	Grain refinement and solid solution
CoCrFeNiMn	40	180 HV → 460 HV	Dislocation density and grain size reduction
AlFeNiCoMn	15	260 HV → 680 HV	Grain refinement, solid solution, and work hardening
AlCoCuNiTi	120	173 HV → 882 HV	Solid solution, lattice distortion, and grain refinement
TiTaVWCr	124	432 HV → 1306 HV	Dislocation density and solid solution

**Table 8:** Crystallite size and BPR values of several HEAs produced by mechanical alloying as well as the hardness and phase evolution of several HEA systems: CrMnFeCoNi [60], CoCrFeNiTi [61], AlCrFeMoNbNi [62], AlCoCrFeNi [63], and AlCoCrFeNiTiZn [64].

HEA Alloy	BPR	Milling Time (h)	Nanocrystallite Size
CrMnFeCoNi	20:1	24 h	~10 nm
CoCrFeNiTi	20:1	5 h	~34–38 nm
AlCrFeMoNbNi	5:1	60 h	~32 nm
AlCoCrFeNi	10:1	24 h	~6 nm
AlCoCrFeNiTiZn	20:1	120 h	~38 nm

**Table 9:** Consolidated HEAs and several mechanical properties.

HEA Alloy	Processing Route [ref.]	Yield Strength (MPa)	Hardness (HV)	Elongation (%)	Reference
AlCoCrFeNi	MA + SPS	1750 MPa	580 HV	2%	[55]
	Casting	873 MPa	362 HV	23%	[57]
	AM (L-DED)	1100 MPa	450 HV	7%	[58]
CoCrFeNiMn	MA + SPS	1534 MPa	425 HV	39%	[56]
	Casting	250 MPa	180 HV	45%	[60]
	AM (LPBF)	600 MPa	260 HV	25%	[61]
Al_0.3_CoCrFeNi	MA + SPS	1400 MPa	480 HV	4%	[62]
	Casting	280 MPa	210 HV	30%	[63]
	AM (SLM)	850 MPa	340 HV	18%	[64]

**Table 10:** HEAs criteria and properties that influence [100].

Criteria	Properties
Mixing entropy	Solid solution formation
Mixing enthalpy	Thermodynamic stability
Valence electron concentration	Phase formation
Electronegativity difference	Phase separation and intermetallic formation
Atomic size difference	Lattice distortion
Melting temperature	Phase stability
Ω parameter	Solid solution prediction
Φ parameter	Thermodynamic stability

## Data Availability

No new data were created or analyzed in this study. Data sharing is not applicable to this article.
